# Analytical Challenges and Metrological Approaches to Ensuring Dietary Supplement Quality: International Perspectives

**DOI:** 10.3389/fphar.2021.714434

**Published:** 2022-01-11

**Authors:** Alessandra Durazzo, Barbara C. Sorkin, Massimo Lucarini, Pavel A. Gusev, Adam J. Kuszak, Cindy Crawford, Courtney Boyd, Patricia A. Deuster, Leila G. Saldanha, Bill J. Gurley, Pamela R. Pehrsson, James M. Harnly, Aida Turrini, Karen W. Andrews, Andrea T. Lindsey, Michael Heinrich, Johanna T. Dwyer

**Affiliations:** ^1^ CREA - Research Centre for Food and Nutrition, Rome, Italy; ^2^ Office of Dietary Supplements, National Institutes of Health, US Department of Health and Human Services, Bethesda, MD, United States; ^3^ Beltsville Human Nutrition Research Center, Agricultural Research Service, US Department of Agriculture, Bethesda, MD, United States; ^4^ Consortium for Health and Military Performance, Department of Military & Emergency Medicine, F. Edward Hebert School of Medicine, Uniformed Services University, Bethesda, MD, United States; ^5^ Henry M. Jackson Foundation for the Advancement of Military Medicine, Bethesda, MD, United States; ^6^ National Center for Natural Products Research, University of Mississippi, Bethesda, MD, United States; ^7^ UCL School of Pharmacy, Pharmacognosy and Phytotherapy, London, United Kingdom

**Keywords:** dietary supplements, food supplements, analytical methodologies, metrological approaches, data management, infrastructures, institutional efforts, case studies

## Abstract

The increased utilization of metrology resources and expanded application of its’ approaches in the development of internationally agreed upon measurements can lay the basis for regulatory harmonization, support reproducible research, and advance scientific understanding, especially of dietary supplements and herbal medicines. Yet, metrology is often underappreciated and underutilized in dealing with the many challenges presented by these chemically complex preparations. This article discusses the utility of applying rigorous analytical techniques and adopting metrological principles more widely in studying dietary supplement products and ingredients, particularly medicinal plants and other botanicals. An assessment of current and emerging dietary supplement characterization methods is provided, including targeted and non-targeted techniques, as well as data analysis and evaluation approaches, with a focus on chemometrics, toxicity, dosage form performance, and data management. Quality assessment, statistical methods, and optimized methods for data management are also discussed. Case studies provide examples of applying metrological principles in thorough analytical characterization of supplement composition to clarify their health effects. A new frontier for metrology in dietary supplement science is described, including opportunities to improve methods for analysis and data management, development of relevant standards and good practices, and communication of these developments to researchers and analysts, as well as to regulatory and policy decision makers in the public and private sectors. The promotion of closer interactions between analytical, clinical, and pharmaceutical scientists who are involved in research and product development with metrologists who develop standards and methodological guidelines is critical to advance research on dietary supplement characterization and health effects.

## Introduction

### Definition

Metrology is the science of measurement and its practice emphasizes an assessment of traceability and measurement uncertainty, concepts that are not always given the attention they deserve in analytical chemistry (King, 1997). It is responsible for the development of internationally agreed upon reference points so that the accuracy, precision, and repeatability of measures of doses or activity can be compared. Metrologists work with research and industry scientists who are making measurements to call attention to standards and to develop and disseminate best practices and new methods.

### Present Context

Dietary supplements (abbreviated as DS throughout this article using the regulatory definitions and framework of the United States) include a variety of ingredients in different countries. They include food supplements and some botanical and herbal medicines in Europe and other regions, listed medicines in Australia, and Natural Health Products in Canada. DS are widely consumed in many countries, purchased not only through brick-and-mortar stores, but also through a variety of online and other marketing channels^1^ (Binns et al., 2018). The products sold often contain dozens of ingredients that vary greatly in their chemical composition, as well as in the information provided about their contents on packaging labels (Dwyer et al., 2018). Both consumers and practitioners expect that supplements contain the ingredients and amounts listed on their labels, and researchers require well-characterized, authenticated products to obtain replicable results. Recommendations and guidance have been issued by regulatory authorities^2^. Nevertheless, when supplements are evaluated, their contents often do not match label claims and quality problems are common, including the presence of prescription drugs, other pharmaceuticals, contaminants (e.g., pesticides, heavy metals, mycotoxins, microbes), misbranded herbal and botanical ingredients, adulterants caused by economic motivations, fillers, dyes, and filth. In other instances, ingredients listed on the label are absent, raising further questions about the quality of DS (Martinez-Sanz et al., 2017).

A great deal of attention was devoted to DS and their sales rose rapidly during the COVID-19 epidemic. Mullin et al. (2021) have recently reviewed commonly used immune-modulating DS, including vitamin D, ascorbic acid, zinc, and melatonin, highlighting the biological plausibility for salutary benefit against COVID-19. However, another recent review on DS in the time of COVID 19 issued by the US National Institutes of Health concluded that currently, data are insufficient to support recommendations for or against the use of any vitamin, mineral, herb or other botanical, fatty acid, or other dietary supplement ingredient to prevent or treat COVID-19^3^.

The safety, quality, and efficacy of supplement ingredients are highly relevant, especially for the more chemically complex DS with beneficial but also adverse health effects (Di Lorenzo et al., 2015; Roytman et al., 2018). They include botanicals as well as products with blends of botanicals and other non-vitamin, non-mineral ingredients and various nutrients. Many such products marketed for sexual enhancement, weight loss, pre-workout, and body building purposes have been found to be spiked with synthetic and often unlicensed drugs. Structure-function claims do not guarantee efficacy or approval by regulatory authorities in the United States^3^ ^,^ ^4^. In Italy as well (Ministero della Salute, 2020), product registration does not correspond to a scientific evaluation^4^ ^,^ ^5^. It is crucial to understand exactly what the claims on DS mean (American Cancer Society, 2021). The claims should be true and not misleading^2^ (Muela-Molina et al., 2021). When they are not, there is good reason for concern about consumer safety (Binns et al., 2018; Crawford et al., 2020). This is particularly true because many adults but also some children are supplement users (Smith et al., 2005; Parnell et al., 2006; O’Brien et al., 2017).

If the analytical problems outlined above, coupled with control and compliance issues, were brought to the attention of the public, consumer and healthcare provider trust in DS would be reduced. Therefore, all stakeholders should pay greater attention to dietary supplement quality through analytical chemistry and better characterization, since these are critical steps in developing and maintaining trust and providing safe, high quality supplement products.

## Objectives

This article discusses the utility of applying rigorous analytical techniques and adopting metrological principles more widely in the study of dietary supplement products and ingredients. With respect to analytical chemistry, some of the newer direct, objective, rapid, and inexpensive quality control methods that could be used internationally to identify ingredients are briefly discussed. The present status of efforts to develop new methodologies and approaches for data analysis, evaluation and management that can be applied to evaluate dietary supplement quality is reviewed, and the need for the techniques and methods to be modified and further improved is examined. A brief discussion of the problems presented by DS follows. The article concludes with case studies of examples involving the analytical characterization and application of metrological principles to botanical DS that has enriched our understanding of the health effects of these products.

### The New Frontiers

At present, the utility of metrology in applying analytical methods with a higher degree of accuracy, sensitivity, standardization and a harmonized system to verify food integrity (Rychlik et al., 2018) is underappreciated, especially in dealing with the many challenges botanical DS present. The new frontiers include opportunities in the development of additional standards, new or better analytical and data management methods, and international engagement and dissemination of these developments.

### Metrology and Dietary Supplement Science

The first new challenge is a need for greater focus of metrology on bioanalytical chemistry and the methods used in studying complex mixtures of ingredients that are often present in dietary supplement research. Metrology has a long and well-established history in physics and engineering, but less so in dietary supplement science. In part, this is because of the lack of validated methods and/or reference standards for the measurement of many dietary supplement constituents. The development of such resources is made challenging by the inherently variable chemistry of many natural products. Heterogeneity in products that share the same general designation (e.g., curcumin), but differ in their chemistry is a recipe for confusion in research involving comparisons between studies, and even more importantly heterogeneity in health outcomes associated with the products (Nelson, et al., 2017; Durazzo et al., 2018; Simmler et al., 2018; Sorkin B. C. et al., 2020).

### Metrology in Data Analysis and Management

The production of reference standards, development of new methodology, and dissemination of best practices in chemical analysis has been the traditional role for metrologists. An emerging challenge is how best to handle and manage the increasingly large amount of data resulting from analytical studies. Often these data are analyzed using modelling, bioinformatics tools, and other software programs. All too often, automated results are accepted uncritically with little attention paid to how the results are generated and how replicable they are. Metrologists have much to offer in assisting analysts in discovering how and why results differ and in quantifying the uncertainty in these measurements (Sene et al., 2017).

### Metrology and International Collaboration

A second new frontier is the need for increased international collaboration on dietary supplement ingredient descriptions (Dwyer et al., 2021). The chemical characterization and quality control of DS and natural health products are important because they easily cross international borders in an increasingly global marketplace. DS manufacturers and analysts in all countries should utilize the resources produced by metrology institutes and the international metrology system to a greater extent than they do at present. This will enable reproducible measurements of DS to be made with a quantified degree of uncertainty. Additionally, metrology institutes should increase their efforts to disseminate their resources and collaborate with DS stakeholders at national and international levels.

Efforts to develop methods and other resources to address quality and safety problems are in progress in several countries. Currently a few bodies such as the European Medicines Agency, AOAC International, the U.S. Department of Commerce’s National Institutes of Standards and Technology (NIST), the National Institutes of Health’s Office of DS (NIH ODS) and the U.S. Pharmacopeia are working in various ways to promote the development and validation of innovative methods to characterize botanicals and other natural products and to disseminate this information internationally. Related groups, such as the food metrology global community are also dealing with many of the same issues^6,^ ^7^. Particularly, the IMEKO^5^ (International Measurement Confederation^5^) has been active. The World Metrology Day was celebrated on 20th May with the theme of Measurement for Health, and in August 2021 the 23rd IMEKO World Congress was held in Yokohama, Japan^8^. Importantly, IMEKO FOODS^9^ was established and is devoted to metrology as it applies to food and nutrition. The first IMEKO FOODS conference was held in 2014 by the ENEA^10^, and the ENEA is currently coordinating a project to build the research infrastructure. It is called “Infrastructure for promoting metrology in food and nutrition” (METROFOOD-RI)^6^. This RI is part of the Health and Food Area of the European Strategy Forum on Research Infrastructure (ESFRI)^11^.

Of course, better regulation and compliance than that which currently exists are also needed (FAO, 2021). It is the regulator’s responsibility to take appropriate steps to ensure that those who produce products respect quality while continuing to improve rigor in new and better methods applications. The absence of an “ideal” *modus operandi* at present should not prevent appropriate regulatory steps to be taken immediately to ensure quality. Communication amongst those engaging in these efforts may facilitate more efficient division of labor and allocation of resources in the future.

## Analytical Challenges and Approaches in Characterizing DS

The rigorous characterization of DS and natural products with accurate, precise, and reliable analytical methods to ensure the reproducibility and quality of preparations is clearly needed to underpin scientific advancement. Metrology has already had a positive impact on DS science through the generation of certified reference materials that anchor analytical results to well characterized standards (Betz et al., 2007).

The major challenges in dietary supplement science with respect to analytical measurements at the metrology interface are providing accurate, reliable, and timely measures of DS quality and applying them to improve dietary supplement quality assessment. Even in the absence of a regulatory or scientific consensus around minimum quality criteria, reference materials and standards can be used in developing and validating analytical methods. This is an important step towards achieving a consensus around understanding and defining quality. Metrology efforts can support those working with botanical supplements by generating a series of botanical reference materials. Matrix-based reference materials for dietary supplement ingredients and products, as well as calibration solutions of key chemical constituents, are powerful tools for making measurements of quality against a regulatory standard or pharmacopeial monograph.

This perspective provides an assessment of current and emerging dietary supplement characterization data analysis and evaluation approaches. This includes both targeted and non-targeted approaches, with a focus on chemometrics, toxicity, dosage performance, and data management for medicinal plants and botanical quality assessment. In addition, statistical methods, and optimized data management methods are considered.

### Analytical Techniques

It is critical to have in-depth knowledge of the major analytical methods in the investigation of “functional” foods and DS (Betz et al., 2018; Spagnuolo et al., 2019). The pros, cons, and data gaps of some conventional and non-conventional techniques are described briefly below, with attention to emerging and innovative analytical methods, including some “green” methodologies.

### Genomic Analyses for Authentication

**FIGURE 1:**
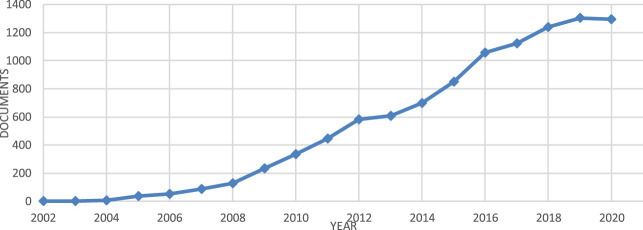
Trends in the number of publications utilizing DNA barcoding (Data from Scopus Database).

DNA barcoding is a taxonomic method using a short genetic marker from a standard part of the genome of an organism’s DNA to identify it as belonging to a particular individual, breed/cultivar, or species (Galimberti et al., 2013; Barcaccia et al., 2016). It has been portrayed as a universal tool that can be linked to any kind of biological or biodiversity information (Galimberti et al., 2013). DNA barcoding systems utilize a short, standardized region (between 400 and 800 base pairs) to identify species (Kress & Erickson, 2012). The technique is based on the assumption that *inter*species variation should exceed *intra*species variation: this difference (i.e. the barcode gap) of a standardized region is exploited for species level identification. This is now a popular method, as revealed by a search in Scopus, with a search for “DNA Barcoding” returning 10,078 publications covering the time period from 2002 to 2020 (see Figure 1).

DNA barcoding may be used to determine whether additional tests for an adulterant or contaminant are needed, but the absence of a signal does not indicate absence of the material originating from that genotype. DNA bar coding cannot be used for authentication except with intact plant material, as standard extracts may not contain intact DNA from the starting material and may contain DNA from excipients.

One goal in the dietary supplement industry is the identification and confirmation of species for processed raw materials that are affected by drying and milling at many points throughout a multilevel supply chain before the finished product is reached. Several studies report on the authentication and identification of plant materials in herbal supplements by means of barcoding. For instance, several reviews have discussed the strengths and limitations in the use of DNA barcoding for identifying botanicals in herbal medicine and DS (Parveen et al., 2016; Raclariu et al., 2018). Further technological advances such as mini-barcodes, digital polymerase chain reaction, and next generation sequencing, genomic skimming approach would provide additional tools for the authentication of herbs and might help in identifying processed ingredients used in finished herbal products (Parveen et al., 2016).

Pawar et al. (2017) compared chemical and DNA barcoding methods when assessing the authenticity of herbal DS. Twelve samples of frequently consumed botanical DS of ginkgo, soy, valerian, yohimbe, and St. John’s wort obtained from the market were evaluated. The results suggested that newer chemical analytical approaches coupled with barcoding or next-generation sequencing (NGS) were promising as a means of analyzing complex botanical supplement products (Ivanova et al., 2016; Haynes et al., 2019; Lo and Shaw, 2019; Handy et al., 2021).

### Nuclear Magnetic Resonance

Nuclear Magnetic Resonance (NMR) spectroscopy is an analytical chemistry technique used in quality control and research for determining the content and purity of a sample as well as its molecular structure. Wu et al. (2020) described the use of chemometric analysis of low-field 1H NMR Spectra for unveiling adulteration of slimming DS by pharmaceutical compounds. Avula et al. (2021) described an integrated workflow for the analysis of bio-macromolecular supplements, including NMR profiling. Simmler et al. (2018) proposed an NMR-based untargeted metabolomic model as a rapid, systematic, and complementary screening for the discrimination of authentic vs potentially adulterated botanicals. Further, Lee et al. (2020) reported a validation study of a proton NMR method for the determination of l-arginine, l-citrulline, and taurine contents in DS.

### Near-Infrared and Mid-infrared Spectroscopy

Infrared Spectroscopy (Small, 1996; Griffiths, and De Haseth, 2007) is “a chemically specific analysis technique that identifies the chemical bonding or molecular structure of materials, based on absorption in the infrared region of the electromagnetic spectrum” (Jadhav et al., 2013). Mid-infrared (MIR) and near-infrared (NIR) spectroscopy techniques combined with chemometrics reflect another emerging procedure, which is being applied in authentication processes to verify if the product contents are in accordance with the label or if it complies with the current legislation.

The fundamental vibrational modes of MIR are in the mid-infrared spectral range (200–4000 cm^−1^), whereas the near-infrared range from 4000 to 13,333 cm^−1^ of NIR contains overtone and combination bands. Sources and detectors in these ranges are required. Spectral dispersion of the signal is normally reached through an interferometric analysis by using a Michelson interferometer. The interferogram is Fourier transformed to yield the spectrum in the frequency domain, which leads to the common name Fourier-transform infrared (FTIR) spectroscopy. The most common instruments are FTIR spectrometers.

Fourier-transform infrared-attenuated total reflection (ATR) spectroscopy is a common and easier to use technique. FTIR spectroscopy represents a rapid, less destructive, and high-throughput method for the analysis of food products, DS and nutraceuticals (Durazzo et al., 2020). It provides simplified handling and enables the samples to be examined directly in their original state. In addition to being more environmentally friendly, FTIR spectroscopy is also a more environmentally friendly procedure, rapid, fast and non-destructive technique, that is simple to perform and requires minimal sample preparation. FTIR spectroscopy is as an innovative analytical technique for determining the “fingerprint” of organic compounds because their functional groups exhibit characteristic signatures in specific infrared regions. Thus, the IR spectra can be used to identify or differentiate between samples. Recent developments and advances in instrumentation as well as chemometric pattern recognition techniques have amplified the range of the IR spectroscopy applications, including evaluating and determining components, monitoring contaminants and adulterants, classification, discrimination, authentication, and other uses. Applications of IR spectroscopy joined with chemometrics are discussed below.

### Liquid Chromatography/Mass Spectrometry and LC/Tandem Mass Spectrometry

Mass Spectrometry (MS) is an analytical chemistry technique used to identify the amount and type of chemicals present in a sample by measuring the mass-to-charge ratio and abundance of gas-phase ions. It is useful for establishing the chemistry of botanical DS (van Breemen, 2020). Over 15 years ago, Sherma, (2003) described qualitative and quantitative analysis by high-performance column liquid chromatography/mass spectrometry (LC/MS) and LC/tandem mass spectrometry (LC/MS/MS) of botanical drugs, drug substances or preparations, and finished botanical products. He also described LC/MS and LC/MS/MS techniques and commercial instruments as well as examples of applications (Sherma, 2003). Currently in the U.S., the NIH and USDA collaborate on the Dietary Supplement Ingredient database (DSID), which provides analytically determined information about the ingredient content in DS commonly sold in the U.S. The analytical contents of vitamins, minerals and botanicals in DS were quantified using HPLC, LC/MS and ICP/MS (Andrews et al., 2017). DS samples were tested along with matrix-matched NIST Standard Reference Materials with known values and uncertainties to monitor laboratory performance. Analytical results were also compared to the United States Pharmacopeia (USP) monographs for multivitamin and mineral content in various dosage forms, monographs for plant extracts and, where available, for finished botanical products (Andrews, Roseland, Gusev et al., 2017). Performance quality standards for dietary supplements should be better harmonized across all major pharmacopoeias: European Pharmacopoeia (Ph Eur), British Pharmacopoeia, USP, and Japanese Pharmacopoeia (JP) (Al-Gousous and Langguth, 2015). Vargas Medina et al. (2020) have more recently described the state-of-the-art and future trends on chip-based LC–MS, including an overview of the commercially available chip-based LC–MS platforms as well as main chip substrates and microfabrication technologies. Rocco et al. (2018) focused on miniaturized separation techniques as analytical methods to ensure quality and safety of DS. LC/MS and LC/MS/MS are extremely sensitive and highly specific techniques for the determination of contaminants and illicit compounds even if present at very low levels.

### Capillary Electrophoresis

Capillary electrophoresis (CE) is a technique used in chemical analysis to separate molecules in an electric field according to size and charge. Gotti (2011) has summarized studies on capillary electrophoresis of phytochemical substances, including alkaloids, polyphenols, carbohydrates, lipids, terpene in herbal drugs and medicinal plants, and provides a short description of the basic principles of capillary electrophoretic techniques. Several previous papers focused on applications of CE toward a specific class of DS or a target biological function. For example, Cianchino et al. (2008) analyzed the potential adulteration of herbal medicines and DS promoted for weight loss by using capillary electrophoresis. Other recent applications of capillary electrophoresis include fatty acid determination in encapsulated vegetable oils supplements (Amorim et al., 2019) and docosahexaenoic and eicosapentaenoic acids determination in marine oil omega-3 supplements (Amorim et al., 2020).

CE with other analytical techniques i.e., mass spectrometry, contactless conductivity detection, evaporative light scattering detection, etc. For example, Restaino et al. (2020) developed a high-performance CE method to determine intact keratan sulfate and hyaluronic acid in chondroitin sulfate samples and food supplements of animal origin. Nguyen et al. (2019) used dual-channeled CE coupled with contactless conductivity detection for rapid determination of choline and taurine in energy drinks and DS. Dos Santos et al. (2016) analyzed amphetamine and its derivatives in “natural” weight loss pills and DS by using CE-tandem mass spectrometry. Duong et al. (2020) proposed using CE coupled with contactless conductivity detection for the determination of 10-hydroxy-2-decenoic acid and free amino acids in royal jelly supplements. CE coupled with evaporative light scattering detection has been used by Bouri et al. (2013) to directly quantitate underivatized amino acids in tea samples.

### Data Analysis and Evaluation

Innovative approaches for data analysis are now available, including sophisticated procedures used in concert with the technologies listed above. Advanced statistical techniques can be applied for data evaluation today. Hypothesis testing and assessment of the likelihood of false positives or negatives are required for analyzing and assessing the significance of “differences” reported for non-targeted or chemometric data. Procedures for dosage design and the toxicity reports as tools for data analysis are also presented in this section.

### Targeted and Non-Targeted Approaches

In the past, the main approaches for quality control of herbal medicines were the ‘component-based’ and ‘pattern-based’ (Mok and Chau, 2006; Zeng et al., 2008). Component-based studies addressed specific characteristic compounds (also known as the marker approach and multi-compound approach), whereas pattern-based studies investigated all detectable compounds (also known as the pattern approach and the multi-pattern approach). Both approaches have limitations. Herbal ingredients used for testing are complex, and single markers do not allow for an adequate evaluation of quality (Simmler et al., 2018). Generally, one or two markers should be considered insufficient for authenticity and quality control of herbs and medicinal plants. More than a few markers are needed to give a total overview of an herbal product and to qualitatively differentiate between products (Simmler et al., 2018).

**FIGURE 2:**
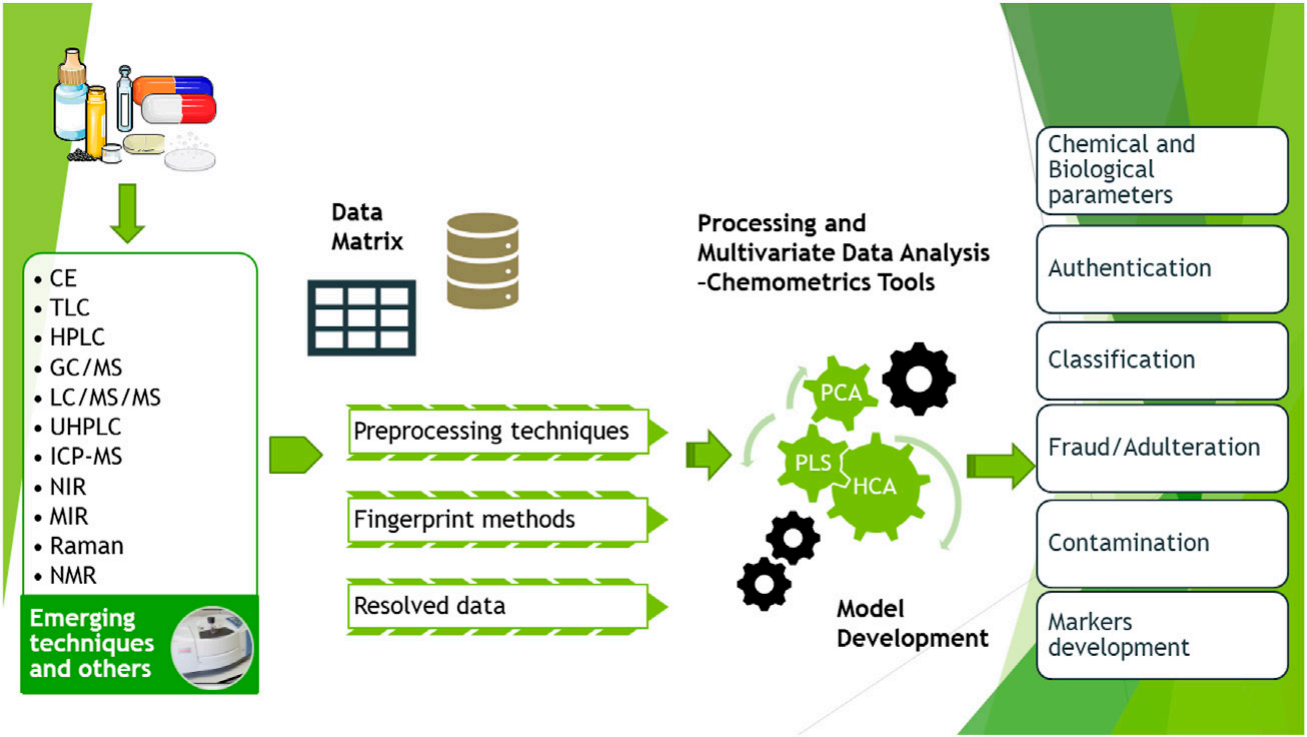
An approach workflow based on fingerprint and chemometrics.

A current strategy is the use of fingerprint analysis and chemometrics. These approaches compare overall chemical compositions (including any bioactive compounds) across multiple samples by using chromatograms acquired from spectroscopy, gas chromatography, liquid chromatography, or mass spectrometry. Figure 2 presents a graphical representation of this new approach covering the workflow for chemistry, manufacturing, quality assessment and controls of botanical drugs.

As shown in Figure 2, the identification of new markers is required and, in this direction, multi-compound, multi-target and multi-pathway studies are being carried out. Harnly et al. (2017) explain how to detect the transition of chemical composition from botanical ingredients to resulting products by means of chemometrics, which permits them to be differentiated quickly. Wallace et al. (2020) gives a current example of identification of adulteration identification in botanical samples with untargeted metabolomics.

Gas chromatography (GC) and liquid chromatography (LC), in conjunction with mass spectrometry (MS), are appropriate for targeted analysis compared to general untargeted analysis; however, results require comparison of full-scan spectra to preestablished libraries (Wu et al., 2012). Using a full screen spectrum reduces the sensitivity of identification of components and may show unknown components. The need for standardization of analytical methods for analysis of dietary supplements is needed, especially methods which are more specific than TLC and LC-UV. Technical difficulties like to the complexity of LC-MS data and annotation of metabolites using untargeted LC-MS is emerging (Chaleckis et al., 2019) and the need for new software tools and a platform that can gather all the existing free mass spectral libraries for metabolite annotation is necessary (Misra, 2021).

Pferschy-Wenzig and Bauer (2015) explored emerging directions in the area pharmacognosy and pharmacological research on herbal medicinal products and suggested that each stage of production should be followed by adequate quality assessment measures (Pferschy-Wenzig and Bauer, 2015). Different methodologies ranging from macroscopic, microscopic, and DNA-based authentication to chromatographic methods i.e., HPLC, TLC, LC-UV, GC–MS and LC–MS and spectroscopic methods i.e., NMR, FTIR-ATR, would be applied, depending on the purpose. The quality of finished preparations would then be evaluated either by means of chemical or bio-marker constituents and/or analytical fingerprints.

### Chemometrics

Hibbert (2016) define chemometrics as “the science of relating measurements made on a chemical system or process to the state of the system via application of mathematical or statistical methods.” Chemometrics uses mathematical and statistical methods to obtain relevant information from collected data (Otto, 2016). Analytical technologies provide complex information. The data treated by chemometrics are often multivariate and such large datasets require multivariate data analytic methods. The statistical techniques are useful for extracting and providing qualitative and quantitative information from complex data, highlighting trends, investigating relationships, building models for defining characteristics of studies and/or for predicting outcomes and drawing conclusions form the experimental data. Chemometrics deal primarily with the extraction of useful chemical information from measured data, rather than theoretical calculations. It has applications in many fields, not only in chemistry (Hibbert, 2016).

The main chemometric pattern recognition techniques are designated as unsupervised and supervised ones. Unsupervised methods aim to identify groups of samples with related features, so they can be separated into different classes. They include principal component analysis (PCA) (Abdi and Williams, 2010), hierarchical clustering analysis (HCA), and partial least squares regression (PLSR). Supervised techniques are designed to extract the information in the mathematical model in order to assign a new sample to an already known class. Supervised procedures include linear discriminant analysis (LDA), PLS-discriminant analysis (PLS-DA), artificial neuronal network (ANN), and soft independent modelling of class analogy (SIMCA), among others.

Chemometrics opened a new scenario for botanicals (Bansal et al., 2014; Durazzo et al., 2018). The chemometric approach is a tool that can be applied in several aspects of the quality control of herbs and medicinal plants used in DS. This can include authentication of individual ingredients, monitoring of the quality of ingredients, identification of chemical constituents, detection of adulteration or contamination, and/or production of standardized formulations. As noted in Simmler et al. (2018), a great advantage of the use of agnostic or non-targeted analyses is that identification of the chemical entities which differ between products or batches is not required, but is facilitated where a novel constituent is associated with biological activity, for example, toxicity.

**FIGURE 3:**
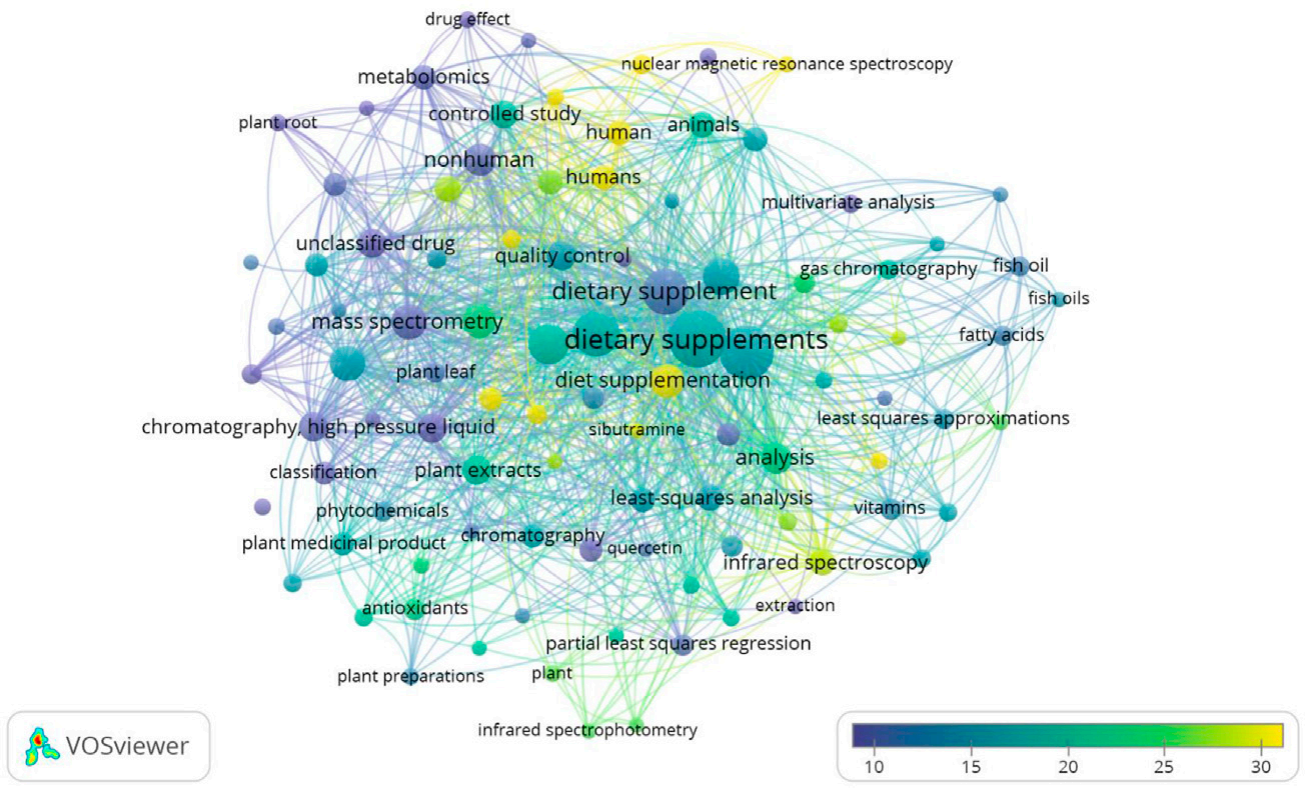
Map of terms for search on: chemometrics and dietary supplement/food supplements research. Bubble map visualizing words from titles, abstracts and keywords of the 111 publications. Bubble size represents the number of publications. Bubble color represents the citations per publication (CPP). Two bubbles are closer to each other if the terms co-appeared more frequently (Based on data from Scopus and elaborated by VOSviewer software).

Figure 3 shows a search conducted on the Scopus platform by using the string “chemometric*” AND “dietary supplement*” OR “food supplement*“. The “full records and cited references” were exported to VOSviewer software (version 1.6.16, VOSviewer software websitewww.vosviewer.com) for further bibliometric analyses and additional processing. The VOSviewer, 2020 software (v.1.6.16, 2020) analyzes the terms/words used in the titles and abstracts of publications, by breaking down the paragraphs into words and phrases, linking them with the citation data of the publications, and visualizes the results in the form of a bubble map by using a term map with default settings (Van Eck and Waltman, 2009; Van Eck and Waltman, 2010; Van Eck and Waltman, 2011; Van Eck, 2011a; b; Waltman et al., 2010). The search led to 111 documents covering a period from 1970 to 2021 with an H index of 22 and 13,18 Citations Per Publication (CPP). A total of 97 terms were derived from the quantitative literature research consisting of 111 publications and depicted as a term map in Figure 3. The top 15 recurring keywords are listed in Table 1. It is interesting to note that “chemometric”, “principal component analysis” and “chemometric analysis” appear among the top 15 keywords, thereby highlighting the integrated approach between analysis and statistics today.

**TABLE 1:** The top 15 recurring keywords on a chemometrics and dietary supplement/food supplements research web search (Based on data from Scopus and elaborated by VOSviewer software).

Term	Occurrence	Total link strength
Dietary supplements	61	746
Chemometric	54	550
Chemistry	41	571
Dietary supplement	41	528
Principal component analysis	30	390
Procedures	27	417
Mass spectrometry	24	327
High performance liquid chromatography	23	324
Chemometric analysis	22	333
Diet supplementation	22	294
Non-human	21	286
Analysis	19	318
Chromatography, high pressure liquid	17	248
Plant extracts	17	237
Quality control	17	247

Examples of infrared spectroscopy joined with chemometrics are described here in greater detail. Deconinck et al. (2019), evaluated the feasibility of using infrared spectroscopy combined with attenuated total reflectance to screen plant-based preparations for nine specific plants (five regulated and four common plants used in herbal supplements). They showed that the best model was obtained with the MIR data by using SIMCA as the modeling technique. They reported that MIR combined with SIMCA could be applied as a first step in the screening of unknown samples, before applying more sophisticated fingerprint approaches or identification tests that are described in several national and international pharmacopeia. In a small study comprising 35 DS on the market for slimming and 34 for male sexual potency enhancements, Deconinck et al. (2017) also applied a strategy based on fingerprinting and chemometrics for the detection of regulated plants.

An example of an investigation that used delayed luminescence combined with PCA is provided by Sun et al. (2019). They proposed simple, direct, rapid, and inexpensive measurements of delayed luminescence for the identifying herbal materials as a first step toward rapid quality control for DS. They also highlighted the importance of developing and establishing a valid and perhaps developing a novel digital tool for the quality control of herbal materials. Sima et al. (2018) used applied electropherograms and chromatograms and chemometric tools, i.e., PCA, cluster analysis and a combination of PCA and linear discriminant analysis (PCA-LDA) and applied them for the authentication of fruit-based extract herbal medicines.

### Dosage Form Performance

Dosage form performance is the ability of a tablet, capsule, or liquid gel capsule to disintegrate and release its functional ingredient(s) into gastrointestinal fluids in a timely manner. Readily disintegrating dosage forms with good release characteristics generally have better oral bioavailability than those with inferior performance profiles. Together, dosage form disintegration and release comprise the process of dissolution. More specifically, dissolution in the context of DS, is an *in vitro* assessment of the rate and extent of phytochemical release into simulated gastric or intestinal fluids using standardized conditions and equipment (Azarmi et al., 2007). Phytochemical dissolution, like that of active pharmaceutical ingredients is a function of a molecule’s physicochemical properties (e.g., molecular weight, polarity, water solubility and their subsequent dissolution into the aqueous gastrointestinal fluids is often greatly diminished, such as when the molecule is hydrophobic. Therefore, inadequate phytochemical dissolution can adversely affect oral bioavailability (Azarmi et al., 2007).

The efficacy of a DS is determined not by only by the active ingredient amount but also the formulation’s design. Supplement formulation can greatly influence the fraction of the ingested bioactive amount that is absorbed, how much reaches the target site within a defined period, and, ultimately, the benefits it might provide for the users. The USP develops performance standards such as *in vitro* disintegration and dissolution tests to detect problems with active ingredient release from dosage forms. The problems may occur due to formulation design and/or manufacturing processes. Disintegration testing demonstrates how a tablet or capsule breaks apart under agitation within 30 min in specific solutions by mimicking gastric or intestinal fluid. A dissolution test measures the amount of a marker compound released from a dosage unit into a stirred solution of simulated gastric or intestinal fluid in a form that is absorbable by the body. A product passes the test if, after 1 h, ≥ 75% of the amount of marker compound is released into the simulated fluid.

Although *in-vitro* disintegration and dissolution tests do not directly predict ingredient absorption and bioavailability (unless such a link has been established for a product experimentally), they are important tools for assuring dietary supplement quality and consistency. Products that failed to pass USP tests may fail to properly disintegrate and release ingredients, and so their active ingredients cannot be absorbed, and expected health benefits may not be achieved. Recently, researchers have shown widespread and inconsistent performance for green tea dietary supplement dosage forms. Based on these results, a recommendation was made to the National Institutes of Health’s National Center for Complementary and Integrative Health policy for “Natural Product Integrity” to consider including satisfactory performance quality of dosage forms as a requirement for funding to avoid inconsistent results in clinical trials due to variability of performance quality (Gusev et al., 2020).

At present, most botanicals do not have published methods in USP for dosage form performance and many existing methods need improvement. DS may have very complex compositions, and there could be more than one option for selecting applicable USP testing protocols. Also, the biological relevance of the hydrodynamics, media, and mechanical stresses in USP disintegration and dissolution testing needs to be evaluated further (Gusev et al., 2020). In this regard, the use of biorelevant dissolution media should be considered. Biorelevant media emulate either fed or fasted conditions by incorporating appropriate electrolytes, enzymes and natural surfactants found in gastric or intestinal fluids in the context of a meal (Dresman, 2014). Since food can markedly influence DS bioavailability, an assessment of dosage form performance in biorelevant media is prudent.

### Data Management

The management of data on dietary supplement quality is of concern nationally and globally because of the enormous expense involved in maintaining and updating very large amounts of data on dietary supplement ingredients and ensuring that the data are valid. Monitoring and surveillance of products is required because many manufacturers in the dietary supplement industry lack transparent methods or adequate audit trails. Many resources are required to maintain and constantly update the increasing number of DS. Dietary/food supplement databases are dynamic and must be constantly updated owing to the frequent changes in the formulation of ingredients and products, introduction of new ingredients and/or possible adulterants.

### Big Data Processing Infrastructures

The term “big data” refers to any data exhibiting unusual features of any of five dimensions: volume, variety, velocity, volatility, and veracity (Fuller et al., 2017). It is important to encourage the use of big data techniques in collection, processing, storage, and analysis to allow for several types of research to go forward, including in-depth investigations of correlations between several types of data at a single point in time.

An example of using big data to deal with knowledge bases relating to DS information is the iDISK, an integrated DIetary Supplement Knowledge base (iDISK). The iDISK integrates and standardizes DS-related information from four existing resources: The Natural Medicines Comprehensive database, the “About Herbs” page on the Memorial Sloan Kettering *Cancer* Center website, the Dietary Supplement Label database, and the Natural Health Products database (Rizvi et al., 2020).

According to Zeb and Soininen (2021) data harmonisation is one of the keys that enable digitalization for foods, and the same methods may be useful for DS. Foods and DS are similar in that a large amount of data is produced; data harmonization produces compatible and comparable datasets consisting of interoperable and widely usable data. Several recent papers (Cade, 2017; Pehrsson, and Mitchell, 2020; Sorkin B. et al., 2020) summarize technologies and new tools and infrastructures for data management, by emphasizing the importance of findability, accessibility, interoperability, reusability of data and related metadata.

Analysis of extremely large “big datasets” computationally may reveal patterns, trends, and associations of interest, such as those relating to human behavior and interactions affecting human health. Cloud-based solutions are on-demand services, computer networks, storage, applications or resources that are accessed via the internet and through a third party’s shared cloud computing infrastructure. The benefits of cloud-based solutions in dealing with big datasets for the end users include increased capacity, scalability, functionality, and reduced maintenance and cost for a computer infrastructure. Some of the newer cloud solutions allow the dynamic allocation of computer resources. They include *Hadoop*, a Java-based, open-source framework for software development that supports the storage and processing of massive data sets, *Spark* is a fast and general engine for large-scale data processing which can quickly perform processing tasks on very large data sets and can also distribute data processing tasks across multiple computers, either on its own or in tandem with other distributed computing tools. The differences between *Hadoo*p and *Spark* technologies are that while *Hadoop* is designed to handle batch processing efficiently, *Spark* is designed to handle real-time data efficiently. *Hadoop* is a high latency computing framework, which does not have an interactive mode whereas *Spark* is a low latency computing and can process data interactively.

Orchestration of the different steps is also key in big data ecosystems. Mansouri et al. (2020) describe automated implementation for performance evaluation of a hybrid cloud for distributed databases that integrate resources between private and public clouds.

#### Hybrid Database Approaches by Using Graph and Relational Databases

Database relationships are associations between tables that are created using join statements to retrieve data. A relational database stores and organizes data points that are related to one another. Based on the relational database model, a relational database presents data sets as a collection of tables and provides relational operators to manipulate the data in tabular form. A distributed database is one database that is one that is spread over different sites, i.e., on multiple computers or over a network of computers that do not need to share the same site or physical components, such as when a database is accessed by various users globally. However, it is managed so that it seems to be a single database to users. Traditionally, relational databases have been used for dealing with dietary supplement ingredients. Data were entered into tables with columns and rows. However, files in such a format are cumbersome, slow, and expensive to manipulate or to join with other databases. When data are likely to need to be connected or joined with other databases, graph databases have the advantage of speedy data retrieval for such connected data. Integrated or hybrid database structure/architecture uses a hybrid of both graph and relational databases and are useful for managing data and improving dietary supplement quality by better dealing with data from integrated approaches. Vyawahare et al. (2018) proposed a hybrid database approach for integrating relational and graph databases in a single system, reasoning that the relational database provided a good structure for storing, managing and querying information, while it was possible to take advantage of the features graph-structured databases as well, thus capturing the strengths of both systems. Structured Query Language (SQL) databases are primarily relational databases whereas “not only” or NoSQL databases are non-relational or distributed database.

Bjeladinovic et al. (2020) proposed an architecture for integration and uniform use of hybrid SQL/NoSQL database components: the SQL managed data contains the fixed or rarely changeable structure, whereas the NoSQL databases are used for vast quantities of data that change rapidly.

## Metrology Applications to Ensure Dietary Supplement Quality: Considerations and Case Studies

This section includes examples of metrological approaches and resources that have been successfully applied to characterize and study several botanical DS in the United States. They serve as an example of how metrology can be used in efforts to improve supplement quality. It could be equally argued that similar approaches are needed in Traditional Chinese Medicine, Kampo medicine or many other systems. The discussion begins with general considerations applying to any chemically complex set of dietary supplement ingredients. It then turns to phytochemical examples while recognizing that many of the same considerations apply to nutrients, other ingredients in supplements, and toxic elements.

The first case study describes the authentication of black cohosh, a commonly used botanical supplement, using a standard reference material. A second describes the measurement of DMAA, a prohibited “natural” substance found in DS. The final case relates the development and fate of one of the first dietary supplement reference materials for complex botanical mixtures produced by the U.S. National Institute of Standards and Technology (NIST). Each of these cases discuss the problems, the potential implications for human health, ingredients measured and what was achieved by the measurement, challenges in quantification and their resolution, and lessons learned. Taken together, they attest to the important role that metrology and analytical chemistry have to play in dietary supplement science.

### Considerations Applying to Measurements of Nutrients and Phytochemicals

The chemical composition of ingredients and formulations used in DS can present considerable challenges for analysts when attempting to separate and measure the chemically diverse nutrients (e.g., vitamins, fatty acids, minerals, phytochemicals) and contaminants such as toxic elements (e.g., Cd, Hg, As, Pb). This significant chemical diversity is especially true for ingredients derived from botanicals and other natural products (e.g., spirulina, fish oils). In this context, it can be very difficult to measure and maintain consistency in the DS products found in the international marketplace and available for study to biomedical researchers.

In response to these chemical measurement challenges, the US National Institutes of Health Office of Dietary Supplements (NIH-ODS) Analytical Methods and Reference Materials Program and the NIST partnered to establish laboratory quality assurance programs (QAPs) with a purpose to promote and support enhanced capabilities for the analytical characterization of DS (Phillips et al., 2011; Sander et al., 2013). These voluntary QAPs, as well as NIST certified reference materials for common dietary ingredients (Rimmer et al., 2013; Eichner et al., 2019; Wise and Phillips, 2019) are designed to help laboratories establish and improve their analytical accuracy, precision, and repeatability of measurements for analytes found in DS. Participant laboratories measure target nutrients and/or phytochemicals, as well as potential contaminants (e.g., pesticides, toxic elements) in samples distributed by NIST, and subsequent data identify analytical challenges, and describe methodological advances.

Through participation in the Health Assessment Measurements QAP^12^ (and in the past the Dietary Supplements Laboratory QAP^13^), industry, academic, and government laboratories from around the world can assess and improve their analytical measurements of botanical dietary ingredients that are popular in supplement products and are of interest to researchers who study dietary supplement health effects. For example, NIH-ODS/NIST QAP exercises have helped the analytical community reduce calibration errors in green tea catechin measurements (DSQAP Ex E; DSQAP Ex I; LG Saldanha et al., 2015), quantify the significance of utilizing a hydrolysis step in the extraction and measurement of soy isoflavones (DSQAP Ex F; Zhang et al., 2015), and validate rigorous methods for the determination of curcuminoids (Mudge et al., 2020).

### Considerations Applying to Investigations of Activities for Chemically Complex DS

Many DS products are chemically complex, in particular those derived from botanicals and other natural products whose chemical compositions are inherently variable, the result of numerous combinations of variables including genetic, environment, and preparative (e.g., harvesting, extraction, formulation) factors. Moreover, the molecular mechanisms of action responsible for their biological effects of interest, including both the specific chemical constituent(s) in the product which contribute to the activity/ies of interest, and their physiological, cellular, or molecular biological target(s), are often unclear (Sorkin B. C. et al., 2020). In some cases, there is a dearth of relevant data, while in others the existing data are heterogeneous. Often rigorous, comprehensive chemical characterization of the materials studied is not available. Documentation of marker compounds may be useful to assess whether the source of a product is likely to be correctly identified, but if characterization relies entirely on a single marker compound it is subject to the following caveats: 1) economic adulteration of the products by addition of a synthetic marker compound can give the appearance that the correct material is present when it is not, or it can deceptively inflate the amount apparently present, 2) in the absence of clear evidence that the marker compound is solely responsible for the biological effects of interest, different products may have identical amounts of marker compound while having very different biological effects, 3) analysis for a single marker compound may fail to detect the presence of adulterants or contaminants.

Agnostic, non-targeted, analytical approaches may address many of these issues with a single method (Urban, 2016). Hight et al. (2019) described a method for computational integration of data from three different high-content, high-throughput analyses (UPLC-MS-MS, gene expression analysis, and detailed cell phenotyping) of the same sets of chemically complex natural product fractions. This allowed the simultaneous generation of strong hypotheses regarding the chemical species contributing to the biological activity and the cellular and molecular substrates through which those compounds exert their effects. Notably, unlike the traditional approach of fractionating a product prior to conducting assays for biological activity, this approach should facilitate the detection of biological activities which require multiple chemical constituents acting on multiple targets, or where multiple constituents are needed for action through the same target. The accompanying UPLC-MS-MS analyses can provide a comprehensive chemical description of the product, as well as of the constituents associated with biological activities of interest.

This approach has human health implications. It can address the problem of contamination or adulteration of products with unexpected compounds through its use of untargeted chemical analyses. Further, by rapidly identifying which constituents are likely to be responsible for the observed activities, the approach facilitates targeted testing of the molecular mechanism of action, in turn setting the stage for future targeted product assessments based on the chemistry critical for the product application or safety.

Although this approach requires a variety of advanced instrumentation and methodology, the US NIH-supported Center for High-Content Functional Annotation of Natural Products is working to develop on-line resources to be used by the community for biological screening, metabolomics and discovery of compound-activity associations^14^.

### Quantification of Metabolites and Human Health Implications

A thorough understanding of DS health effects requires a knowledge of dietary intake and chemical composition as well as the resulting metabolite profiles present in clinical samples. To advance understanding of their health implications, the NIH-ODS/NIST Health Assessment Measurements QAP approach is unique in its pairing of studies that measure the chemical composition of dietary ingredients/supplements representing human intake with measurements of nutrient, phytochemical, and toxic metabolites in serum, plasma, or urine. These clinically focused QAP studies are designed to promote the measurement of physiologically relevant analytes in addition to measurement of traditional biomarkers that may or may not have a known biological activity.

Many methodological resources available can help research scientists and industry analysts measure myriad constituents in DS. A major issue, however, is that established good practices in analytical chemistry and pharmaceutical sciences may not be uniformly accepted or applied within the larger dietary supplement scientific community. It is important that rigorous and fit for purpose methods be applied to support the development of domestic and international standards, regulations, and legislation.

### Case Studies

#### Case Study 1: Authentication of Black Cohosh Using the National Institutes of Standards and Technology Standard Reference Material

##### Challenges in the Use of Black Cohosh

Advances in metrology science have traditionally been passed to researchers in the form of certified reference standards. These standards allow verification of in-house quality control standards and assure the accuracy of quantitative analytical results world-wide. However, qualitative analysis, specifically authentication of botanical supplements, introduces new demands on standards that are not necessarily in agreement with the intended purpose of certified reference materials. Metrology standards still have a useful role in validating the chemical components of supplements, but a lesser role in authentication.

Authentication of botanical supplements is most effectively accomplished using non-targeted methods in conjunction with pattern recognition programs (chemometrics) such as principal component analysis (PCA) and partial least squares-discriminant analysis (PLS-DA). Of primary importance is establishing the natural range of biological variation of a species or sub-species. Using these approaches it is possible to establish if a sample is abnormal (adulterated, contaminated, or substituted) without knowing what is normal and the range of normality.

Establishing normality and its range is usually accomplished by accumulating authentic materials that encompass all the expected variables such as genetics, environment, management, and processing. An appropriate collection of authentic materials can be analyzed and then used to build a model and to determine or whether an unknown sample is authentic (lies within the model) or different (lies outside the model). Questions regarding how many authentic samples are necessary, what variables to cover, and what method of analysis to use are critical and beyond the scope of this case study and addressed elsewhere (LaBudde and Harnly, 2012).

In 2014, a study was initiated to develop a method for authentication of black cohosh (*Actaea racemosa*) (Harnly et al., 2016). For this study, four species of vouchered, authentic *Actaea* samples (*A. dahurica*, *A. pachypoda*, *A. podocarpa*, and *A, racemosa* (black cohosh) were obtained from four sources and four black cohosh candidate standard reference materials (SRMs) were obtained from the National Institute of Standards and Technology (NIST). Samples were analyzed by flow injection mass spectrometry (FIMS), nuclear magnetic resonance spectrometry (NMR), and DNA barcoding. This case study presents the FIMS results for the four sets of authentic black cohosh samples analyzed by principal components analysis (PCA) and soft independent modeling of class analogy (SIMCA).

Samples were acquired from the American Herbal Pharmacopoeia (AHP, Scotts Valley, CA, United States), North Carolina Arboretum Germplasm Repository (NCAGR, Asheville, NC, United States), Strategic Sourcing, Inc (SSI, Banner Elk, NC, United States), and NIST (Gaithersburg, MD, United States).

##### Results

**FIGURE 4:**
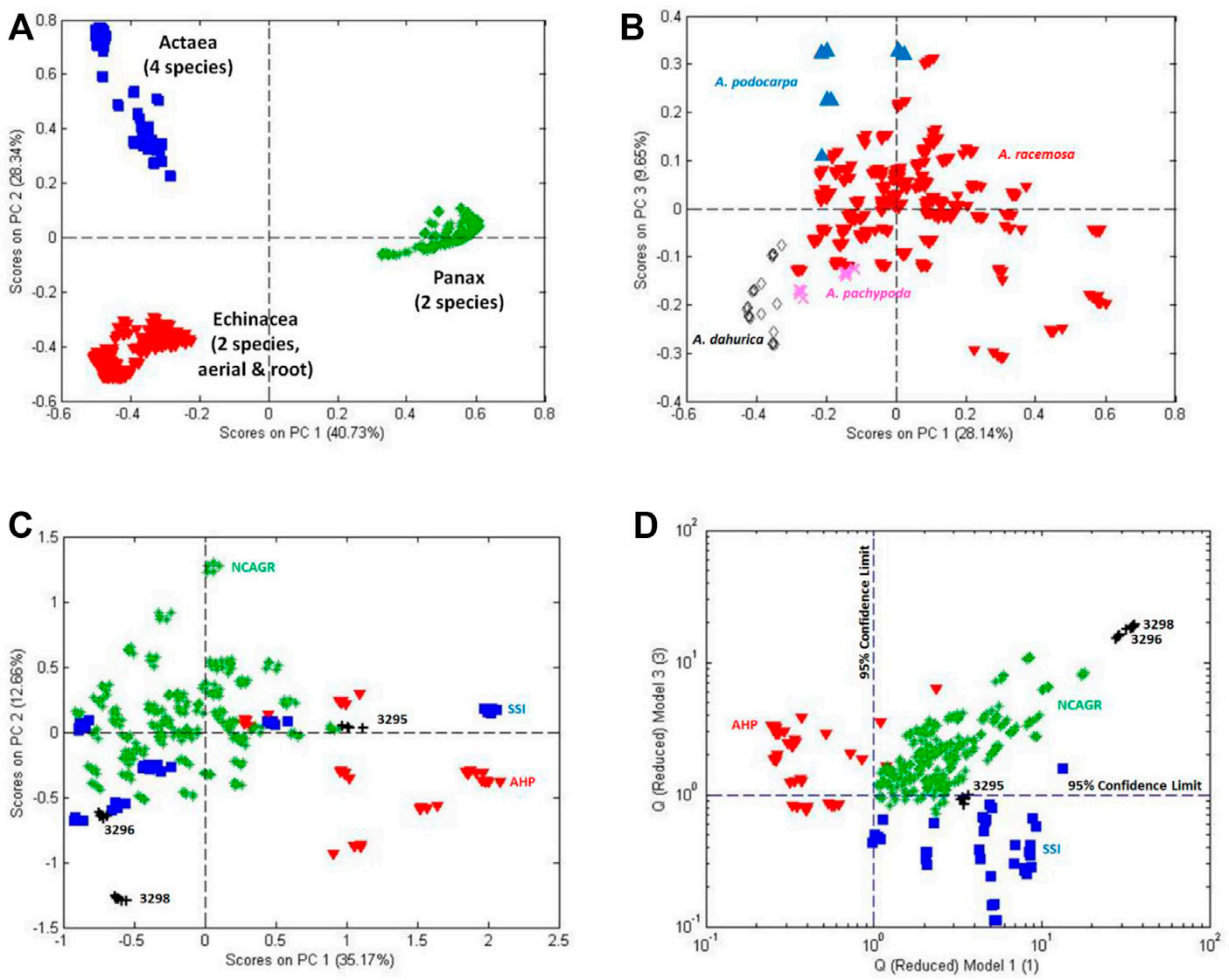
Authentication of Actaea racemosa: **(A)** PCA score plots of three genera (*Actaea*, *Echinacea*, and *Panax*), **(B)** PCA score plot of four species of Actaea (*A. dahurica, A. pachypoda, A. podocarpa*, and *A. racemosa*), **(C)** PCA score plot of vouchered A. racemosa rhizomes from three sources (American Herbal Pharmacopoeia, North Carolina Arboretum Germplasm Repository, and Strategic Sourcing, Inc.) and NIST SRM 3295 (rhizome), 3296 (leaves), and 3298 (extracted dosing form), and **(D)** Double SIMCA Q residuals plot for the same samples shown in plot C.

Figure 4A provides perspective by showing the PCA score plot for three genera: four species of *Actaea* (*A. dahurica*, *A. pachypoda*, *A. podocarpa*, and *A. racemosa*), two species (*E. purpurea* and *E. angustifolia*) and two plant parts (aerial and rhizome) of *Echinacea*, and two species of *Panax* (*P. ginseng* and *P. quinquefolius*). The three genera were easily separated and identified by FIMS.

The 2-dimensional PCA score plot for four species of *Actaea* (Figure 4B). The 2-dimensional plot suggests the four species can be separated and identified. Discrimination at the 95% confidence limit was verified by SIMCA (data not shown).

Figure 4C shows the PCA score plot for authentic black cohosh materials from the four sources listed above. Samples from NCAGR (green) were rhizome materials hand-collected from 22 sites in the eastern US. AHP (red) and SSI (blue) each collected and vouchered seven rhizome materials from un-identified sources. The NIST SRMs were obtained from an unidentified commercial company producing a black cohosh supplement. The company provided rhizome (SRM 3295), leaf (SRM 3296), extracted rhizome (SRM 3297), and extracted solid dosing form (SRM 3298) samples. Each sample was analyzed 4 times.

Each organization provided authentic, vouchered black cohosh samples. The PCA score plot was expected to show all rhizome samples in a homogeneous cluster with only the leaf material (SRM 3296) and the extracted solid dosing form (SRM 3298) lying outside the cluster. The rhizome and leaf, different physical parts of the plant, were anticipated to have different compositions and the extracted solid dosing form would have lost and gained components during the preparation process.

The PCA score plot in Figure 4C, however, shows relatively clear separation of the AHP and NCAGR rhizomes with the SSI samples spread between the two clusters. Further SIMCA studies (Figure 4D) confirmed the difference in the chemical composition of the authentic black cohosh samples. Figure 4D is a complicated double SIMCA plot showing the residuals for independent models of the AHP (*X* axis) and SSI rhizomes (*Y* axis). The AHP rhizomes are primarily located to the left of the vertical 95% confidence limit and are significantly different from the other rhizomes. The SSI rhizomes are located below the horizontal 95% confidence limit and are significantly different from most of the other rhizomes. The NCAGR rhizomes to the right and above the 95% confidence limits can be judged as significantly different from the other rhizomes.

The NIST rhizome standard (SRM 3295) can be grouped with either the SSI or NCAGR rhizomes but appears statistically different from the AHP rhizomes. In general, however, the carefully collected and processed NIST rhizome standard (SRM 3295) falls roughly in the middle of the other vouchered rhizome samples while the leaf (SRM 3296) and extracted dosing form (SRM 3298) are distant from the rhizomes in the upper right corner. Thus, despite the variation between authentic samples, SRM 3295 validates their identification as black cohosh.

The failure of the rhizome samples from the different sources to form a single cluster can be attributed to a number of factors such as growing location, harvest year, handling, preparation, or storage conditions. With respect to growing location, a detailed examination of the NCAGR samples showed distinct compositional differences between some of the samples from the 22 growing locations (Harnly et al., 2016). Differences were attributed to soil, weather, endophytes, and local genetic variations. Unfortunately, there is a lack of similar data (metadata) from the other sources.

These data serve to illustrate two major points. First, there is considerable compositional variation between all plants (indeed, all living organisms) despite their morphological similarity. This variation must be accounted for by any model that seeks to establish authenticity. Second, a reference standard from a metrology organization can validate the identification of the genus, species, and plant part. This case study shows that, although a single NIST SRM could not provide information regarding the biological, environmental, and processing variability, it did confirm the accuracy of the non-targeted mass spectral profile.

#### Case Study 2: DMAA: A Prohibited “Natural” Substance

##### Challenges With DMAA

1,3-dimethylamylamine (also methylhexanamine or DMAA) is an amphetamine derivative added to dietary supplement products with claims of being a “natural” stimulant derived from geranium (Cohen, 2012; Eliason et al., 2012), although there is no conclusive evidence to support the claim of being from a botanical (Zhang et al., 2012). DMAA, promoted for weight loss, bodybuilding, and performance enhancement, was first introduced as a drug for nasal decongestion in 1948 before being voluntarily removed from the market in 1983. In 2006 it was re-introduced in 2006 as Geranamine, an extract of *Geranium* and constituent of geranium flower oil. In 2013, the U.S. Food and Drug Administration (2004) stated that “DMAA is not a dietary ingredient, and DMAA-containing products marketed as DS are illegal and their marketing violates the law.” (U.S. Food and Drug Administration, 2018). Nevertheless, despite the FDA ruling, DMAA continues to appear in dietary supplement products offered on the market.

##### What Is Measured and what Is Achieved

Approximate concentrations of DMAA in popular DS range from 0.11 to 673% of what is noted on the label, and products typically provide between 4.6–25 mg in a single serving. This wide range may reflect differences in extraction/analysis methodologies, insufficient quality control in dietary supplement production, as well as shifting formulations (Austin et al., 2014). In fact, inconsistencies were noted between ingredients listed on product labels and those found within the product when analyzing products by using analytical methods: some products also contain DMAA despite it not being listed on the Supplement Facts label (Cohen et al., 2017). Further, some DS contain DMAA in combination with other stimulants and ingredients either included/excluded on the label or, which could further amplify the stimulatory effects of DMAA. The serving size of DMAA is also not always specified on the label, particularly when included as part of a proprietary blend. As such, without a clear understanding of the presence of DMAA and in what amounts, it is difficult to be certain what effects, if any, can be attributed to DMAA (McCarthy et al., 2011; Powers, 2015). Regardless, DMAA is not a DS ingredient and the marketing of products with DMAA according to the FDA “…violates the law”. The only way to understand what is in any product is through applying quality testing and analysis.

##### Problems With the Quantification of Metabolites and Human Health Implications

As a synthetic stimulant and vasoconstrictor, DMAA narrows blood vessels and arteries and increases blood pressure, which could lead to cardiovascular (e.g., heart attack, shortness of breath, arrhythmias, tightening of the chest, heart attack), neurological, and psychological problems. In fact, numerous case reports have linked DMAA-containing products to several serious adverse events including liver injury, cardiac arrest, stroke, brain hemorrhage, and death (following physical exertion) (Eliason et al., 2012; Col et al., 2013). Animal toxicology studies, moreover, have shown that the systematic toxicity of DMAA in animals is greater than ephedrine, another restricted stimulant that is not only linked to several side effects but can also lead to abuse and addiction (Palamar, 2010).

Scientific research concerning the safety of DMAA-containing products is limited at best given the regulatory framework for DS. In addition, DMAA is not a legitimate dietary ingredient. Based on the available literature, serious adverse events may be due to consuming quantities of DMAA and what can be tolerated by human consumption, both of which are actually unknown (Schilling et al., 2013). In some case reports describing significant reactions, the person had ingested almost 15 times the DMAA serving size reported on the label in addition to other stimulants (Gee et al., 2010; Gee et al., 2012). Only a handful of DMAA pharmacokinetic studies have been conducted in humans, and these suggest only a fraction of an orally administered DMAA dose is even metabolized (Bloomer et al., 2018). Because DMAA is often combined with other ingredients, the concomitant administration of other ingredients (e.g., caffeine) may inhibit DMAA clearance and lead to excessive concentrations (Bloomer et al., 2018). This remains to be demonstrated and has not been confirmed through analytical methods.

##### Resolution of Issue/Problem

Given the numerous reports of serious adverse events associated with DMAA-containing products and concerns about potential abuse, as noted above, the FDA declared DMAA a potential health risk and illegal for use as a DS ingredient in 2013 (U.S. Food and Drug Administration, 2018). DMAA is currently banned by multiple countries (e.g., United States, Ireland, Sweden, Denmark) and organizations (e.g., World Anti-Doping Agency, Department of Defense’s Operation Supplement Safety) (Cohen, 2012; Col et al., 2013; Operation Safety Supplement, 2021).

The FDA continues to identify and remove DMAA-containing DS products from the market as quickly as possible by either giving companies the opportunity to voluntarily recall and destroy products or, in instances of non-compliance, administratively detaining and/or seizing products. Despite being prohibited since 2013, however over 268 products on the market in 2021 clearly listed DMAA on the Supplements Facts label even though it is illegal for use in DS (Crawford and Deuster., 2020).

As the Food and Drug Administration FDA continues its efforts to remove these violative products from the market, consumers should not buy or use any product containing DMAA. Because some retailers still have stocks of discontinued or reformulated products with DMAA, consumers should carefully read the product’s Supplement Facts label to verify it does not contain DMAA. Although DMAA is most commonly listed as 1,3-dimethylamylamine; 2-amino-4-methylhexane; dimethylpentylamine; geranamine, or methylhexanenamine, it can also be noted under several different names, including but not limited to 1,3-DMAA; 1,3-dimethylpentylamine; 2-hexanamine, 4-methyl-(9CI); 4-methyl-2-hexanamine; 4-methyl-2-hexylamine, or; dimethylamylamine. Further, some products that list *Pelargonium* graveolens extract or *Geranium* extract may also contain DMAA and should be reviewed with caution; a full list of the various terms and synonyms for DMAA can be found on the Operation Supplement Safety website^15^ ^,^ ^16^. Despite these efforts, these products continue to be on the market, whether the consumer knows it or not. Metrology and analytical methods can pre-emptively establish what is contained in these products and avoid dangerous or misleading labels from entering the marketplace. Such methods should be established and implemented for DMAA.

For now, consumers are also encouraged to check OPSS for a list of current, discontinued, and reformulated products noting DMAA on their product label/website. Consumers should be encouraged to use only DS that have evidence of third-party testing, which confirms the product’s label ingredients^17^.

##### Lessons Learned and Other Comments

Numerous case reports have documented adverse events associated with DMAA containing products (Eliason et al., 2012, Department of Defense 2013). Studies that have reported its safety, moreover, may be biased as they were supported by companies responsible for marketing DMAA-containing supplements (Zovico et al., 2016). Although some confusion about DMAA’s classification as a natural vs synthetic simulant has emerged, the no reliable, definitive evidence substantiaties DMAA’s status as being derived naturally from a botanical; DMAA should not be sold as a DS ingredient. Finally, most studies on DMAA are based on results gathered from products that include other ingredients in addition to DMAA. Research to investigate the safety of DMAA as a single ingredient is not available.

Rigorous study designs developed to evaluate the safety of DMAA, especially in populations with concomitant use of other substances and high frequency of use, are needed to understand the magnitude of the association between DMAA and serious adverse effects. Until then, because it is difficult to ensure their safety, DMAA-containing products should continue to be prohibited.

#### Case Study 3: Development of Ephedra Standard Reference Materials in the United States

##### Challenges Associated With Ephedra

*Ephedra sinica* (Ma huang) is the only extant genus of gymnosperm shrubs in its family (Ephedraceae) and order (Ephedrales). In DS, ephedra (ES) is usually either a formulation of powdered stems and aerial portions or a dried extract (Natural Medicines Database, 2018). The plant contains multiple chemical compounds, but the major effects of ES are likely caused by ephedrine (Ibragic and Sofić, 2015); although possibly other bioactives are also contributors. The principal alkaloid constituents are ephedrine, pseudoephedrine, and sometimes small amounts of phenylpropanolamine along with smaller amounts of other constituents (e.g. norpseudoephedrine, methylephedrine, and norephedrine and tannins) (Gurley et al., 2015). Ephedrine is also sold as a drug used in stimulants in the 1930s and World War II, and for weight loss in the 1970s. During the late 1990s to mid 2000s ES was included a dietary ingredient usually in combinations of ephedrine alkaloids, caffeine and other phytochemicals in widely popular over-the-counter DS advertised primarily for weight loss and sports performance in the United States and Canada.

##### Identification and Characterization

Assays for ephedrine and related alkaloids were available in the 1970s and 80s, but the analytical models were mostly titrations, not suitable for measuring alkaloid in complex multi-botanical finished products. It woud have been difficut to assess the amounts in DS and compare them against a standard.

The amounts and constituents in ES containing DS varied greatly, not only in ephedrine but in many other alkaloids (Natural Medicines Database, 2018). According to the Natural Medicines database, in studies for the treatment of obesity, the typical dose of ES was 15–40 mg of the ES alkaloids, calculated as ephedrine, taken up to three times daily. In some studies, this dose of ES was taken with caffeine 100–210 mg daily in divided doses. In other studies, other combinations were used that included 12 mg of ES with 40 mg of guarana three times daily plus 17 other vitamins, minerals, damiana, bee pollen, Ma huang and other ingredients and 60 mg of ES daily with cola nut and willow bark (Natural Medicines Database, 2018). Different brands used different amounts in their DS products. For the larger brands sold commercially, batch to batch uniformity of the same brand was usually good, while for the smaller brands that used the herb rather than the extract results were less consistent. Labeled amounts were generally fairly accurate because the products were often spiked with ephedrine alkaloid, and determination was by quantities added. There was little demand for ephedrine alkaloid reference materials in DS. By the late 1970s pharmaceutical scientists clearly recognized the potentially adverse effects of certain formulations of ES products seemed to be greater when combined with caffeine (Gurley et al., 2015). Therefore, reference materials with the inclusion of caffeine were needed. Also, DS also existed in several matrices, each of which required different reference materials.

##### Issues and Human Health Implications

Reports from Europe about the efficacy of ephedrine as a prescription weight loss drug stimulated great interest in the nutrition community and public, ES products sold over the counter without prescription had the potential for being used to treat obesity based (Astrup et al.,. 1985). US law views DS as foods so they are considered inherently safe, unlike drugs for which benefit and risk are weighed. However, during the early 1990s ES use became increasingly associated with adverse side events that were troubling, including drug interactions, blood pressure and circulatory problems (stroke, fainting, heart rhythm disorders) and depression. By the early 2000s private individuals and attorneys general in various states around the country were bringing many lawsuits against manufacturers. Also, state public health authorities attempted to limit the sale of products containing large amounts of ephedrine. However, federal law required clear evidence of harm before a DS product could be seized or banned. Compelling evidence was needed because the legal climate was anti-regualtory, and in the 1990s US Food and Drug Administration prosecutors had lost several important agency cases in federal courts and FDA’s regulatory powers were subsequently restricted. FDA’s original response to ES related adverse events was publication in 1997 of proposed rule embodying a regulatory approach od limiting the dose and permitting ES-containing products to remain on the market while limiting their total alkaloid amounts (Porter 2007). Thus FDA needed reference standards so that when the contemplated ceiling on the amounts of ephedrine that woud be allowed in DS products, manufacturers and regulators would have a common standard to use for assessing compliance. Other agency efforts to mitigate risk under DSHEA that had been used successfully for other ingredients continued as well. These included a 2001 FDA request for voluntary recall of aristolochic acid (AA) containing ingredients by manufacturers and distributors coupled with an import alert on AA containing botanical dietary ingredients. FDA posted warnings for consumers about pyrrolizidine alkaloids in comfrey in 2001. Also in 2001 FDA warned health care practitioners that kava might be a serious health risk It accompanied the warning with a request for them to review and report cases of adverse effects. Congress continued to be supportive of the Dietary Supplement and Health Education Act (DSHEA) of 1994, and so a Congressional action to ban ES was unlikely. In the early 2000s the Secretary of the Department of Health and Human Services had the ability to declare ES an imminent hazard, but this step was not taken, and FDA lawyers were concerned about losing another regulatory battle. To make an adulteration charge, FDA needed evidence (Porter 2002). However, in the face of DSHEA’s new provisions, there was still uncertainty about the required level of evidence on the dangers to human health of ES containing DSs that would be conclusive enough for FDA to remove products from the market. Therefore, until regulators deemed the adverse events and scientific evidence sufficient to defend FDA’s action to completely remove ES containing products from the market, FDA continued to pursue what might be a practical and politically acceptable way to minimize harm to users by regulating the amount of ephedrine in the supplements. This effort was difficult to implement technically because the limit on total alkaloids pushed the limits of analytical reproducibility for the minor alkaloids. Also, FDA’s adverse event data did not indicate that a predictable dose/response existed.

At the same time there was a growing public outcry over a number of high-profile ES associated deaths. In 2001, the NIH’s Office of Dietary Supplements (ODS) received Congressional language encouraging it to enhance clinical research on the safety and efficacy of DSs. This move was spurred by concerns over the increasing use of ES and ephedrine for weight loss and athletic performance and the deaths. ODS sponsored an Agency for Healthcare Research on Quality (AHRQ) review conducted by the Rand Corporation’s Southern California Evidence-based Practice Center, and it was completed in 2003 (Agency for Healthcare Research on Quality 2003; Shekelle et al., 2003a) The review concluded that DS containing ES/ephedrine (usually in combination with caffeine) had modest short-term effects on weight loss, although long term effects on weight were unavailable and unknown. Ephedrine plus caffeine was associated with a boost in immediate physical performance for fit young men. However, there was no evidence that ES or ephedrine improved long-term physical performance of athletes or that it would do so for most members of the public. ES and ephedrine increased the risk of nausea, vomiting, jitteriness, and palpitations. Moreover, there were more serious risks linking the products to catastrophic events such as sudden death, heart attack, or stroke. While individuals with a history of cardiovascular disease, those taking high doses of ES containing supplements, and those taking it in combination with other stimulants such as caffeine were be expected to be at increased risk, some of the severe adverse events occurred in individuals with no preexisting medical problems, those taking relatively low doses of ES, or taking ES alone (Shekelle et al., 2003b). Although, when the study was first published it was still unclear whether these data were sufficient to remove the products from the market, the RAND evidence-based review contributed needed information for generating the FDA ban on ES in DS in February 2004.

##### Resolution of the Problem

Metrology became involved early dealing with ES. The initial 1997 FDA dose limiting regulatory approach for mitigating the harms caused by ES DSs was hampered because analytical resources (methods, and matrix reference materials) were insufficient to support a proposed rule (Betz et al., 1997; Hurlbut and Carr 1998). NIST resolved the problems involving lack of reference materials necessary for enforcing possible regulations involving ceilings for the amounts of ephedrine in DS by beginning development of a suite of ES reference materials. Many cases of heart problems and some deaths linked to ES containing DS continued to be reported, but public outcries and calls for action mushroomed after the death of a sports celebrity, the 23-year-old Baltimore Orioles baseball pitcher Steve Belcher, from a stroke after taking diet pills containing ES.

Although pure ephedrine alkaloids for use as calibration standards had long been commercially available from fine chemical companies, NIST’s contribution of matrix reference materials with certified values for ephedrine alkaloids and caffeine was critical. The reference materials were intended primarily for methods validation and use as control materials to support the analysis of DS and related botanical materials. In 2006 a suite of five ES-containing dietary supplement Standard Reference Materials (SRMs) was issued by the National Institute of Standards and Technology (NIST) with certified values for ephedrine alkaloids, synephrine, caffeine, and selected toxic trace elements. The materials represented a variety of natural, extracted, and processed sample matrixes that provided different analytical challenges. The content of constituents was determined by multiple independent methods with measurements performed by NIST and by three collaborating laboratories. The methods utilized different sample extraction and cleanup steps in addition to different instrumental analytical techniques and approaches to quantification. (Sander et al., 2005). Because ES containing supplements were frequently produced as blends with other ingredients which appeared to enhance cardiovascular effects (e.g., caffeine and other botanicals), values for these were also included in the reference suite (Brown et al., 2007).

However, by 2006, since FDA had banned ES in DS in 2004, reference materials for quality control centering on dose to limit the amounts of actives in marketed products were no longer needed. By the time the methods and materials became available, the regulatory goal had changed to developing methods suitable for enforcing a limit of zero tolerance (Roman 2004; Sharpless et al., 2006). Since ES was banned shortly after the development of the NIST reference materials. they were never used as much as was originally intended. In the first years after the 2004 ban the furor against ES was so great that at one point, officials at the US Drug Enforcement Agency who feared that the matrix reference materials might be purchased by unscrupulous individuals and diverted to produce methamphetamine requested NIST to stop selling them.

Soon after the 2004 ban of ES containing products, “ES free” weight loss products began to appear in increasing numbers. The first was bitter orange (also called Seville orange or sour orange), which contains synephrine, the main bioactive in ES, as substitutes for ES. NIST developed a standard reference material (SRM) suite for Bitter Orange which provided three forms representing different challenges analytically: ground fruit, extract, and a solid dose oral table form. The SRM provided certified concentration values for synephrine, octopamine, tyramine, N-methylytramine, hordenine, total alkaloids and caffeine.

##### Lessons Learned and Other Comments

The first lesson learned from the ES experience was the important role of metrology in improving the quality of complex botanical DS like ES, caffeine and bitter orange containing DS products by providing certified SRM to validate measurements of the major bioactives. This stimulated the later development of a new category of SRM for DS by NIST (Sander et al., 2006). These SRM are used primarily in method development, as control materials, and to assist manufacturers of DS in characterizing raw materials for potency, authenticity, and contamination or adulteration. SRM are also valuable in assisting in assessment of consistency and quality in finished products and can be used by the DS industry and measurement experts to improve DS quality and ultimately reduce public health risks that may be associated with such products (Sander et al., 2006).

A second lesson was that CRM are helpful in improving quality *only* if they are used. A DS industry with higher quality standards ideally would lessen safety risks. Although some large DS manufacturers use the CRM and call for stronger quality enforcement, other DS producers place a low premium on improving DS product quality. There was little regulatory incentive to improve quality under current law and enforcement measures, but in recent years the FDA has issued Good Manufacturing Practice guidance for DS that may be helpful.

The third lesson is that although ES SRMs were not used as widely as was originally intended, the experience amassed in developing them has been helpful in development of other SRM for DS. Since 2006 NIST has issued SRM for dietary supplement matrices including: ES, bitter orange, *Ginkgo biloba*, saw palmetto, St. John’s wort, green tea, yerba mate, kelp, turmeric, ginger, multivitamin/multielement tablets, botanical oils, and fish oils. All are characterized with values assigned for the content of active ingredients and/or marker compounds and toxic elements. Two experts predict that in the next decade the focus in development of food and DS CRMs will be on nutrients in food and DS matrices and use of isotope dilution (ID) LC-MS and ID LC-MS/MS methods for the determination of vitamins and other organic nutrients, all with matrices with potentially lower (<3%) expanded uncertainties. (Wise and Phillips 2019). From a metrological standpoint, the values assigned for all vitamins in food- and DS-matrix CRMs should be determined using MS-based methods with ID quantification, if feasible, rather than with microbiological assays. DS-matrix SRMs now have values assigned for over 80 organic and inorganic nutrients, toxic elements, proximates (e.g., protein, fat, moisture, ash and carbohydrates), and contaminants.

A fourth lesson is that only a small number of DS containing ES or ephedrine alkaloids at low doses are still being sold on the US market today compared to the higher doses and larger number before the 2004 ban although new threats to the public health emerge when a product is banned. In 2021 the NIH Dietary Supplement Label database listed only 94 products with mentions of ES on the label and 15 contained varying amounts of ES extracts. ES in DSs was banned in the European Union (EU) in 2006, and it is illegal in the United States, Australia and Canada and several other countries. ES was ‘in the marketplace’, mostly as *Ma Huang*, pre-DSHEA, and that herb is still sold in traditional Chinese pharmacies and also appears in some foods. It was “grandfathered in” by law and not subject to FDA’s new drug ingredient (NDI) notification rules.

A fifth lesson is that irrational DS product formulations that fly in the face of pharmaceutical science are still being marketed by manufacturers who fail to seek the advice of knowledgeable scientists who could warn against using potentially risky combinations of multi-ingredient botanical products with caffeine and other chemicals. Premarket clinical testing and approval are not legally required and so their adverse effects may go undetected. More evaluation of these complex mixtures and their physiological effects is needed to protect the health of the public. In spite of the ES experience, DS of complex botanical mixtures with serious adverse cardiovascular effects became popular after the ES ban and are still being marketed for weight loss, sports performance and energy enhancement. The tablets and pills are devoid of ephedrine alkaloids but containing bitter orange, with the synephrine usually present as a natural part of the plant, and less frequently as the synthetic form, or as a purified form from the plant. In one study, concentrations varied from 5 to 14 mg/g in five different DSs (Santana et al., 2008). The amounts of the caffeine containing ingredients often exceeded a serving of caffeinated beverages) and other botanicals with many different pharmacological activities were present in addition to the *Citrus aurantium* extracts. The product labels made it difficult to determine exactly how much caffeine was present, since it was often in proprietary blends or only the botanical sources were listed (Gurley et al., 2014). More recently, DS and “energy drinks” containing very high amounts of caffeine have also been associated with cardiovascular problems (Institute of Medicine 2014; Gurley et al., 2015). Although little evidence exists that bitter orange is safer than ES, many products are sold with it listed as an ingredient, usually as an extract. In 2021 the DSLD listed 474 product labels with bitter orange as an ingredient, usually as an extract, and some also contained caffeine although they probably contained lower amounts of both ingredients than in the early 2000s.

All that has been learned from the unfortunate history of ES containing DS does not guarantee that another DS might not cause serious health problems in the future. The United States lacks a registration system for DS and continues to allow “natural” botanical sources of caffeine, ephedrine alkaloids and propanolamine combinations in DS. Since the early 1980s FDA has restricted the marketing of their synthetic forms in “amphetamine look-alikes” because of the cardiovascular risks they impose (Gurley et al., 2015). FDA focuses on safety risks but monitoring the myriad products now on the market exceeds their resources. DS product labeling for non-nutrient ingredients is confusing because the amounts of these bioactives are not required to be listed in a standard manner by DSHEA and also many are in proprietary blends, which do not disclose amounts. Some of these problems created by DSHEA remain and legislators have not yet revisited the law and resolved the issues Until the law changes, it is unlikely that such information will be disclosed.

## Recommendations

### More Involvement of Analytical Chemists Pharmaceutical Scientists and Metrologists in Research on Dietary Supplement Quality

This article has focused on expanding the role of analytical chemistry and metrology in assuring dietary supplement quality and safety to the extent that the bioactive dose affects it. Guidelines for evaluating herbs, medicinal plants and other ingredients using these tools should be disseminated more widely.

Clearly, metrology and a comprehensive and accurate understanding of the chemistry of complex products are necessary first for quality, which is key to beginning the process of addressing the safety and efficacy of products for replicable research. However, the safety and efficacy of DS also depends on issues involving design, replicability, and interpretability of clinical trials (especially of botanical supplements). These are additional important matters that need attention but are outside of the scope of this paper (Sorkin B. et al., 2020). Metrologists must become familiar with these issues and work with other experts to deal with them.

### Greater Awareness of the Regulatory and Communications Context of Methods Development, Metrology and Analysis

Metrologists and analytical chemists working in the field of DS can maximize their impact on public health if their plans are designed to be relevant to regulators both at home and abroad. More communication is needed between, metrologists and analytical chemists and pharmacovigilance experts within and between countries on ingredients and new compounds such as excipients that while safe in drugs consumed over a few days may not be so in supplements consumed over weeks or years, that may be causing adverse events. Finally, there is the issue of international communication which at present appears to be on an ad hoc basis rather than through regular interactions between experts in various countries. While the current efforts of various agencies (AOAC International, US Pharmacopoeia, the European Medicines Agency, and others) to disseminate information on dietary supplement ingredients internationally are helpful, the advisability of launching complementary efforts to optimize outcomes is appealing. The US NIH’s National Center for Complementary and Integrative Health (NCCIH) regularly updates its guidance on compliance with its natural product integrity policy, and both NCCIH and the ODS now have formalized policies to help ensure appropriate characterization and reproducibility of reagents and experimental interventions used in funded research^18^. This policy establishes guidance on the information required by ODS and NCCIH for different types of products used in both mechanistic and clinical research including complex botanical and animal products, probiotics, refined products, and placebos. Importantly, the NIH Policy notes that multiple, orthogonal analytical methods may be needed to appropriately characterize natural products and DS, noting that techniques to confirm the authenticity of a product “…may include, but are not limited to, HPLC, UV, MS, IR, NMR, and should, when taken together, generate a unique profile, or “fingerprint,” which can be diagnostic for that substance”. However, although research grants involving DS are now reviewed for product integrity, many federally funded studies still appear to lack rigor in this regard in the United States.

### Communicate Findings to Other Disciplines Nationally and Internationally

Another area requiring further work is in the dissemination of findings. In contrast to well established channels for disseminating analytical methods internationally, the communication of findings about dietary supplement quality to the public is usually confined within national boundaries and are not widely publicized internationally. Broader dissemination may be in order since many Web e-marketing ventures involving problematic DS often bypass some or all country-level regulatory control efforts. Although some valuable informal and irregular “cross talk” goes on among some of the pharmacovigilance experts across countries more regular communication may be in order. Metrologists, pharmacists, toxicologists, natural product chemists, pharmacognosists, and food and nutrition scientists could work more effectively with regulatory authorities and others to disseminate their findings both nationally and globally. Present efforts to deal with regulatory problems and international communications and recommendations for enhancing them will be addressed in a future communication.

### Conclusion

Awareness is increasing on the importance of analytical replicability and rigorous chemical characterization in DS, and powerful methods are now available for such characterization. However, metrology is often underappreciated and underutilized in addressing the many challenges presented by complex botanical DS. Greater use of metrology resources and expanded application of metrology approaches are needed, and current best practices should be disseminated more widely. These steps will ensure that academic researchers, regulators, and scientists in industry are familiar with and apply metrology in addition to other analytical resources to improve the quality, safety, and effectiveness of DS. A new frontier for metrology is promoting interactions between the analytical, clinical, and pharmaceutical scientists who are making products and conducting research with metrologists to develop standards and establish methodological guidelines. This is a critical step to advancing research on DS. A second new frontier for metrology is in developing improved analytical methods, standards and data management methods in research on DS and communicating these developments to academic and industry researchers and analysts, as well as to decision makers in the public and private sectors. Finally, more international collaborative efforts are needed to speed the development of internationally agreed upon measurements that can enhance the basis for regulatory harmonization, support reproducible research, and advance scientific understanding.

## Footnotes

1
https://www.rakutenintelligence.com/blog/2016/online-vitamin-sales-growing-faster-rest-e-commerce.

2
https://www.fda.gov/food/dietary-supplements-guidance-documents-regulatory-information/dietary-supplement-labeling-guide https://www.fda.gov/food/dietary-supplements-guidance-documents-regulatory-information/dietary-supplement-labeling-guide-chapter-iv-nutrition-labeling; https://www.teknoscienze.com/Contents/Riviste/PDF/DIET_SUPP_AF4_2013_RGB_16-17.pdf; http://www.ahpa.org/Portals/0/PDFs/Policies/Guidance-Documents/AHPA_Herbal_Supplements_Labeling_Guide.pdf.

3 Dietary supplements in the time of Covid19 https://ods.od.nih.gov/factsheets/COVID19-HealthProfessional/.

4
https://www.fdareader.com/blog/tag/structure+function+claims.

5
http://www.salute.gov.it/portale/temi/p2_6.jsp?lingua=italiano&id=3668&area=Alimenti%20particolari%20e%20integratori&menu=registri.

6
https://www.imeko.org.

7
http://www.metrofood.eu/.

8
https://imeko2021.org/.

9
https://www.imeko.org/index.php/tc23-homepage.

10
https://www.enea.it/en.

11
https://www.esfri.eu/.

12
https://www.nist.gov/programs-projects/dietary-supplement-laboratory-quality-assurance-program.

13
https://www.nist.gov/programs-projects/health-assessment-measurements-quality-assurance-program.

14
https://hifan2.sites.ucsc.edu.

15
OPSS.org.

16
https://www.opss.org/sites/default/files/downloadable/OPSS%20DS%20Products%20Containing%20DMAA_508.pdf.

17
https://www.opss.org/article/dmaa-prohibited-stimulant.

18
https://www.nccih.nih.gov/research/nccih-policy-natural-product-integrity.
